# Focal radiotracer uptake in the falciform ligament; A rare lymphoscintigraphic pattern in breast cancer

**DOI:** 10.22038/aojnmb.2024.80086.1564

**Published:** 2025

**Authors:** Amin Saber Tanha, Farid Jafari Zarrin Ghabaei, Pegah Sahafi, Mohammad Ahmadi, Ramin Sadeghi

**Affiliations:** Nuclear Medicine Research Center, Mashhad university of Medical Sciences, Mashhad, Iran

**Keywords:** Breast Lymphoscintigraphy, Falciform Ligament, Breast Cancer

## Abstract

Breast cancer lymphoscintigraphy is a crucial tool in pre-operative assessment, typically revealing sentinel lymph node drainage patterns within axillary and extra-axillary regions. However, rare cases challenge conventional understanding. We report a 67-year-old woman with breast cancer, where lymphoscintigraphy revealed focal uptake within the falciform ligament of the liver, an exceedingly rare phenomenon. Clinical examination and imaging showed no axillary lymph node involvement. Lymphoscintigraphy and subsequent Single-photon emission computed tomography (SPECT)/computed tomography (CT) uncovered two axillary lymph nodes and an atypical focal uptake in the falciform ligament. Histopathology revealed no metastasis in sentinel nodes. The conventional understanding of breast lymphatic drainage primarily involves axillary and extra-axillary pathways, with the falciform ligament rarely implicated. This case suggests a unique lymphatic pathway connecting the breast and liver, possibly influencing metastasis. Factors such as lymphatic obstruction and valvular incompetency may contribute. This case highlights the importance of comprehensive lymphatic mapping in breast cancer evaluation and underscores the need for further research into atypical lymphatic pathways.

## Introduction

 Breast cancer lymphoscintigraphy, a pivotal tool in pre-operative evaluation, typically reveals sentinel lymph node drainage patterns within the axillary and extra-axillary regions. 

 However, in rare instances, atypical lymphatic pathways challenge conventional understandings. 

 Herein, we present a case of a 67-year-old woman diagnosed with breast cancer, where lymphoscintigraphy unveiled a distinct focal uptake within the falciform ligament of the liver, an exceedingly rare phenomenon in breast cancer lymphatic mapping.

## Case Presentation

 A 67-year-old woman presented with a palpable mass in the left breast, subsequently diagnosed as invasive ductal carcinoma through core needle biopsy. Clinical examination and ultrasound evaluation revealed no evidence of axillary lymph node involvement. The patient was referred to our center for pre-operative lymphoscintigraphy to facilitate sentinel lymph node biopsy. Lymphoscintigraphy was conducted using a single injection of 37 MBq of [^99m^Tc]Tc-Phytate in 0.1cc volume in the supero-lateral border of the left breast peri-areolar area. Post-injection planar imaging conducted one hour later unveiled two distinct foci of activity in the axillary region.

**Figure 1. A:**
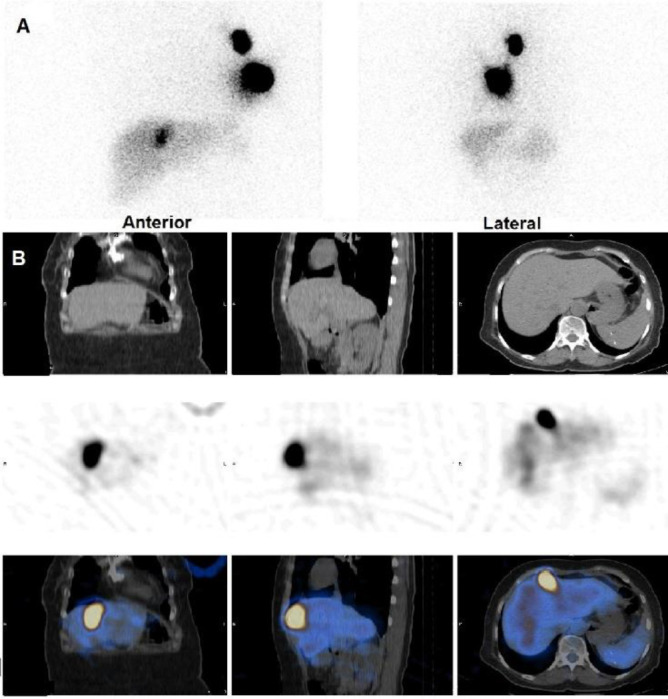
The static images show a focus of increased tracer activity in the left axillary region as well as another focus of increased tracer activity in the zone of liver. **B**. The SPECT/CT images confine the focus of increased tracer activity in the hepatic region to the falciform ligament with no underlying lymph node

 Additionally, an atypical focal radiotracer uptake was observed in the anterior view in the background of diffuse liver uptake (Figure 1A). 

 Following acquisition of the initial planar images, the patient underwent single-photon emission computed tomography (SPECT)/ computed tomography (CT) for precise localization of the focal uptake in the hepatic region, revealing alignment with the falciform ligament. Nevertheless, no distinct underlying lymph node was discerned along the ligament (Figure 1B). Histopathologic evaluation of the sentinel nodes at the level I of the left axilla on the subsequent day's surgery revealed an absence of metastatic involvement.

## Discussion

 The conventional understanding of breast lymphatic drainage primarily encompasses pathways towards the axillary and extra-axillary regions, encompassing the internal mammary, lateral and medial intramammary, as well as supraclavicular regions (1). However, the identification of a route for breast lymphatic drainage via the falciform ligament is exceedingly rare. The falciform ligament, serving to demarcate the hepatic lobes and tether the liver to the anterior abdominal wall, notably harbors lymphatic vessels that are presumed to facilitate superficial liver lymphatic drainage towards the supra diaphragmatic nodes, including the anterior diaphragmatic and internal mammary lymph nodes(2). This intricate lymphatic pathway establishes a junction point between breast and hepatic lymphatic drainage, potentially contributing to the rare occurrence of breast cancer metastasis to the liver or vice versa (3–6). Factors such as lymphatic obstruction because of the tumoral involvement of axillary or extra-axillary sentinel nodes in breast cancer and valvular incompetency of falciform lymphatic vessels or impaired lymphatic contractility are postulated as key contributors to lymphatic backflow from the breast to the liver via the falciform ligament (7). 

 In the presented case, the absence of metastatic involvement in sentinel nodes and the early detection of focal falciform uptake prompt consideration of incompetent lymphatic contractility or defective lymphatic valves within the lymphatic vessels(8).
